# Adherence to Anti-Malarial Treatment in Malaria Endemic Areas of Bangladesh

**DOI:** 10.3390/pathogens12121392

**Published:** 2023-11-27

**Authors:** Mohammad Sharif Hossain, Mohammad Abdul Matin, Nur-E Naznin Ferdous, Anamul Hasan, Saiful Arefeen Sazed, Amit Kumer Neogi, Sumit Chakma, Md. Atiqul Islam, Afsana Alamgir Khan, Md. Ekramul Haque, Shayla Islam, Md. Nazmul Islam, Wasif Ali Khan, Md. Akramul Islam, Rashidul Haque, Mohammad Shafiul Alam

**Affiliations:** 1 Infectious Diseases Division, International Centre for Diarrhoeal Disease Research, Bangladesh (icddr,b), Dhaka 1212, Bangladesh; mshossain@icddrb.org (M.S.H.); abdul.matin@icddrb.org (M.A.M.); atiqul.islam@icddrb.org (M.A.I.); wakhan@icddrb.org (W.A.K.); rhaque@icddrb.org (R.H.); 2 Bangladesh Rural Advancement Committee (BRAC) Health Programme, BRAC, Dhaka 1212, Bangladeshamit.kn@brac.net (A.K.N.);; 3 Directorate General of Health Services (DGHS), Ministry of Health and Family Welfare, Government of Bangladesh, Dhaka 1212, Bangladeshnimunna@yahoo.com (M.N.I.)

**Keywords:** malaria, drug adherence, adherence, compliance

## Abstract

Ensuring adherence to antimalarial treatment is crucial for achieving a radical cure and elimination of malaria, especially in hard-to-reach areas. We conducted this study to assess the current scenario of drug adherence in four endemic sub-districts of Bangladesh. Among 110 enrolled participants, 70% were mono-infected with *Plasmodium falciparum* and the remaining 30% with *P. vivax*. The overall treatment adherence frequency was 92.7% (95% CI: 83.0–96.3%). A total of eight participants were found to be nonadherent to treatment and all of them were from Bandarban. Level of nonadherence was equally observed in two age groups: 11–17 and 18+ years. However, male participants (*n* = 6) were found to be more nonadherent than females (*n* = 2). Among 7.3% with nonadherence to treatment, a single participant with *P. falciparum* mono-infection refused to take medication and became nonadherent. Remaining participants stated that they were feeling well and going to work, thus leaving treatment course uncompleted. Although overall compliance with malaria medication seems good, a gradual increase in noncompliance to *P. vivax* malaria treatment suggests that the National Malaria Elimination Program must be enhanced and monitored to fulfil the projected malaria elimination goal before 2030 from Bangladesh.

## 1. Introduction

Malaria is transmitted by female *Anopheles* mosquitoes infected with five species of *Plasmodium* parasites*—Plasmodium falciparum*, *P. vivax*, *P. malariae*, *P. ovale*, and *P. knowlesi* [1,2,3]. The high rate of mutation, dynamic life cycle, and rapid genome replication of *Plasmodium* spp. Allow them to adapt quickly [4,5]. Among 13 districts with malaria endemicity, Rangamati, Khagrachhari, and Bandarban from Chittagong Hill Tracts (CHT) are hyper-endemic, contributing more than 90% of total malaria case burden in Bangladesh [6]. *P. falciparum* is the main malaria parasite causing human infection in Bangladesh, followed by *P. vivax* [7,8]. But mixed infections by both of the species with some scattered reports of *P. malariae* infection were reported as well [9,10]. In comparison with 2008, the mortality and morbidity of malaria in Bangladesh has fallen by 93% and 94%, respectively, in 2020. However, the prevalence of *P. vivax* infections increased from 5% to 20.3% between 2011 and 2020 [6], which was further increased to 32.1% in 2022 (unpublished NMEP data).

According to the current national guideline for malaria treatment in Bangladesh, artemether–lumefantrine combination (AL) for three days with a single dose of primaquine (PQ) on the first day is recommended as the first-line of treatment for infections caused by uncomplicated *P.-falciparum-*infected malaria with a provision for other alternative artemisinin-based combination therapies (ACTs), such as artesunate–amodiaquine, artesunate–mefloquine, and dihydroartemisinin–piperaquine [11,12]. On the other hand, the treatment of *P.-vivax*-infected malaria is administered as a three-day course regimen of chloroquine (CQ) along with primaquine (PQ) to be given from the first day for 2 weeks [13]. The emerging artemisinin resistance and corresponding treatment failure in Vietnam, Cambodia, Thailand, and neighbouring Myanmar raise the potential threat for malaria control in other endemic areas like Bangladesh [14,15,16,17,18,19]. Apart from the treatment failure and drug resistance, recurrence poses a major threat to the elimination of malaria in *P. falciparum* and *P. vivax* co-endemic areas as well [20,21,22].

The success of drug effectiveness largely depends on high levels of patient adherence to antimalarial treatment [23]. Patients’ adherence to the antimalarial drugs is an important factor to determine the therapeutic response and parasite resistance [24,25,26]. The majority of the malaria-endemic areas in Bangladesh are located in hilly and forested regions, which pose difficulties for timely communication and prompt access to treatment. Maintaining high adherence to treatment becomes even more crucial in those areas. Nonadherence to treatment can lead to recurrent episodes of malaria to the same individual and contribute to the development of drug resistance against antimalarial medications [27,28].

Since 2007, the unyielding and continuous support from the Global Fund to fight AIDS, Tuberculosis and Malaria (GFATM) has paved the way to control malaria and achieved remarkable success in Bangladesh. The joined collaboration between the government of Bangladesh and different nongovernmental organizations (NGOs) consortium led by the Bangladesh Rural Advancement Committee (BRAC) has been implementing this GFATM-funded malaria elimination program in malaria endemic districts [29]. Despite some successful initiatives like the distribution of long-lasting insecticide-treated nets (LLIN) for vector prevention and rapid diagnostic test (RDT) for prompt malaria diagnosis in the community, there are several gaps and difficulties that could potentially prevent the National Malaria Elimination Program (NMEP) from achieving its current goals. There is neither any documented evidence of ACT treatment failure as of yet nor any report on treatment adherence to ACT that has been available since 2007 in Bangladesh [30]. In addition, no studies have been conducted to assess whether patients are adequately completing the prescribed 14-day course of Primaquine to eliminate *P. vivax* hypnozoites. The emergence of antimalarial drug-resistant strains in neighbouring countries underscore the need to assess patient adherence to drug and identify the influential factors. Therefore, this study aims to estimate the current level of adherence to antimalarial drugs, in accordance with national guidelines, within malaria hyperendemic areas of Bangladesh.

## 2. Materials and Methods

### 2.1. Study Setting and Population

The study areas were selected based on recommendations from NMEP. Bandarban has the highest malaria incidences in Bangladesh, accounting for roughly 56% of the country’s overall incidence in 2019 [31], which has increased to 76% in 2022 (NMEP unpublished data). Therefore, three sub-districts (Alikadam, Thanchi, and Lama) from Bandarban with the highest annual parasitological index (API) and Chakaria from Cox’s Bazar with low API were selected for this study. Study participants were enrolled from 1 October to 31 December 2020, covering different ethnic groups from Murong/Mro, Marma, Tripura, Tongchangya, Chakma, and Kuki/Usai along with Bengali descendants. The malarial mono-infection of every eligible participant was confirmed by the RDT and/or microscopy from the corresponding Upazila Health Complex (UHC), community clinics, and in community by the health workers.

### 2.2. Sample Size

Assuming an adherence rate of 85% with 7% precision rate, 95% confidence interval, and 10% loss to follow-up, a total of 110 patients were required for this study [32]. Eligible study participants of 11 years of age and above as well as having been administered with treatment for malaria mono-infection with *P. falciparum* and *P. vivax* were enrolled. According to NMEP data, there was 12–35*% P. vivax* infection in the study areas, so we hoped to enrol at least 30 participants with corresponding mono-infection in our study.

### 2.3. Adherence Definition

Adherence was assessed using questionnaires with varying levels of detail regarding how and when medications were taken (self-report); physical counts of remaining tablets in blister packages or dispensing envelopes (pill counts); pill boxes with electronic caps that track the date and time of each opening; biological assays; and combinations of these techniques [23]. In our study, the pill count method was adopted in measuring adherence.

Following the interview and verification of the quantity of remaining pills, the patients enrolled in the study were labelled as either probably adherent or probably nonadherent or definitely nonadherent. If the participants reported having taken all the prescribed medications within the correct time period and had no remaining pills, they were labelled as probably adherent. Otherwise, they would be probably nonadherent if they reported not taking the full course of medication within the prescribed time duration and dosage but failed to display any remaining pills. Those who displayed any leftover pills were defined as definitely nonadherent [28].

### 2.4. Sampling Strategy

**Figure 1:**
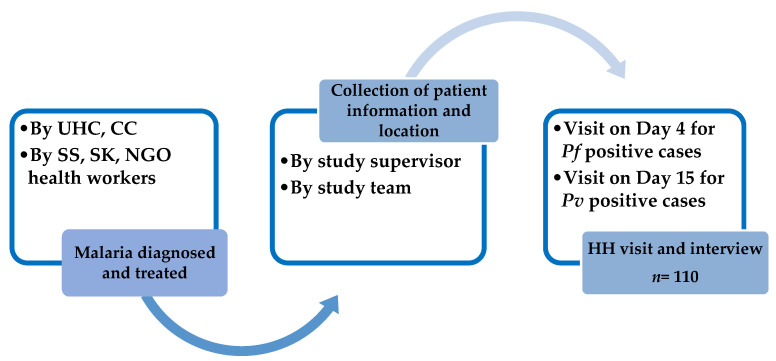
Schematic diagram of sampling strategy for the enrolment of study participants.

During the visit to the corresponding UHC, the contact addresses of prospective eligible patients were collected by the study team. Additionally, information was gathered from community health workers, such as BRAC Shasthya Sebika and/or NGO consortium field workers working under the NMEP platform. Household visits were conducted either on day 4 for patients who received antimalarial treatment for *P. falciparum* infection or on day 15 for those who received treatment for *P. vivax* infection. The selection process was carried out using a first-detected, first-enrolled approach until a total of 110 participants were reached (Figure 1).

### 2.5. Data Collection and Analysis Procedures

A semi-structured questionnaire was developed to obtain information from the participants. Data were entered into SPSS version 20.0 (SPSS Inc., Chicago, IL, USA) and any data that did not meet prespecified developed queries were excluded from the analysis. The statistical analysis was performed using Stata version 15.1 (Stata Corporation, College Station, TX, USA). Descriptive statistics were used to analyse the baseline characteristics of participants/caregivers. Two groups—probably nonadherent and definitely nonadherent—were combined into the nonadherent group [33,34]. The adherence between *P*. *falciparum* and *P. vivax* was compared using *t*-test and bivariate analysis was conducted with Chi-square or Fisher exact test. A *p*-value of <0.05 was considered to be statistically significant.

### 2.6. Ethical Approval and Informed Consent

This study was approval by the institutional Ethical Review Committee of icddr,b (Protocol no: PR-20097 and date of approval: 25 September 2020). Participants or their legal guardians were asked for informed written consent/assent before being enrolled in the study. For participants aged between 11 and 17 years, verbal assent was obtained as well. Extensive discussions were conducted with all participants, ensuring that they were fully informed about their right to discontinue the interview at any time, without the need to disclose their reason for withdrawal or fear any negative consequences.

## 3. Results

### 3.1. Demographic Characteristics

Among 110 participants, 70% of them experienced *P. falciparum* (*Pf*) mono-infection (*n* = 77) and the remaining 30% with *P. vivax* (*Pv*) during the time of the study. A majority of the patients (*n* = 99, 90%), regardless of their mono-infection with *Pf* (*n* = 71, 92.2%) or *Pv* (*n* = 28, 84.1%), came from three sub-districts of Bandarban. The baseline demographic characteristics of all study participants are provided in Table 1. The majority of them were male (*n* = 84, 76.4%) and 18 years and above (*n* = 73, 66.4%). The most represented ethnic background was Mro (*n* = 42, 38.2%), which was followed by Bengali (*n* = 33, 30%) and Tripura (*n* = 20, 18.2%) descendants. Among the participants, farming with traditional jhum cultivation was the main occupation (*n* = 42, 38.2%). On the other hand, just shy of 70% of participants had either no formal education or had just passed primary level. About three quarters of the participants (*n* = 80, 72.2%) belonged to families with five or more members.

### 3.2. Level of Adherence

Since eight participants were found to be nonadherent, the overall adherence rate was 92.7% (95% CI: 83.0–96.3%). The levels of nonadherence (NA) were 3.9% and 15.1% with *Pf* and *Pv* mono-infection, respectively (Table 2).

The level of adherence was further stratified into age group, gender, and demographic locations (Table S1). Unlike age group, where level of nonadherence was equally observed in (11–17) and (18 and above), male participants (*n* = 6) were more nonadherent than females (*n* = 2). Meanwhile, all nonadherent cases were observed in Bandarban.

### 3.3. Reasons for Nonadherence

Among 7.3% participants with nonadherence to treatment, their primary reason for nonadherence was feeling well and going to work, thus leaving treatment courses uncompleted. Every nonadherent participant to *Pv* treatment (*n* = 5) and all but one to *Pf* treatment (*n* = 2) indicated this reason for nonadherence. A single participant with *Pf* mono-infection refused to take medication and became nonadherent.

## 4. Discussion

Nonadherence to the antimalarial medications poses a potential threat to the emergence of drug resistance undermining the progress achieved so far. Furthermore, there could be some serious socio-economic repercussions if the achievement of the radical cure against *P. falciparum* and *P. vivax* would fail. Patient adherence is directly related to the importance of early diagnosis and prompt and effective treatment in malaria endemic regions [35]. Unlike other studies conducted elsewhere [24,36], this was the first ever attempt to demonstrate such an investigation of adherence to both *Pf* and *Pv* antimalarial treatments in real-life settings in Bangladesh, as far as we know. The overall adherence was found to be satisfactory (93.1%). Like a similar study conducted more than a decade ago in Bandarban [30], adherence to *Pf* medication was observed to be higher (95.8%) in our study compared to neighbouring countries—Myanmar (89.5%) and Sri Lanka (73.8%) [37,38].

The failure to comply with PQ treatment can prevent the effective elimination of gametocytes, leading to relapses [39]. Gametocytes of *P. vivax* have the ability to form dormant liver stages know as hypnozoites, which can be reactivated weeks to months after the initial infection [22], even after the parasite having cleared from the bloodstream [40]. Despite attaining gradual progress towards disease elimination, with an overall decrease in malaria incidence, there has been a consistent increase in *P. vivax* infection since 2014 in Bangladesh [6]. This might be attributed to the comparatively higher noncompliance with *Pv* treatment, particularly with regard to PQ. Our findings concordantly reported the adherence for treating *P. vivax* was lower than the corresponding adherence observed in *Pf* (95.8%). Although compliance rate to *Pv* treatment (84.9%) was similar to other study conducted in Brazil (86.4%) [28] and higher than some other malaria endemic countries like India (15.3%) [41] and Venezuela (76.3%) [42], the treatment adherence for *P. vivax* infection should be particularly demanding and closely monitored.

Several factors related to socio-economic, education, and environmental aspects contribute to the low adherence to antimalarial treatment [28,43,44]. In our study, the primary reason for nonadherence was the perception of feeling well before completing the full course regimen. There might be another reason behind higher adherence to *Pf* treatment. Adherence is higher for shorter treatment plan like a 3-day treatment regimen with ACT or 7-day treatment with PQ, thus seconding the statement that the longer the treatment duration, the lower the adherence or compliance to dose regimen, according to other studies [27,28,45].

In spite of having more male participants in the study, female counterparts showed higher treatment adherence, as previously observed [46]. Unlike another study where children, adolescent, and elderly populations were more prone to adherence [47], no such association between the age of the participants and treatment compliance for *P. falciparum* was observed. In this study, the adherence level for *P. falciparum* was found to be higher (*n* = 29, 96.7%) among 11–17 years, which was noteworthy considering other regions. For example, AL treatment was administered among a 5–14 years age group with 89% adherence level in Uganda [33]. In Ethiopia [48] and Malawi [49], levels of adherence, however, were 52.5% and 60.6% among the 5–17 years age group, respectively.

This study has some limitations. Pill count for measuring adherence is not always considered as the most accurate technique. The sample size was too small to meaningfully report on *Pv* cases alone. So, the data regarding nonadherence among participants with *Pv* mono-infection would not be suitably robust to report. There were neither any data on single low-dose PQ among *Pf* patients nor 3-day CQ regimen among *Pv* patients because those patients were out of the scope to be evaluated in this study. Finally, recruitment from Thanchi was limited due to its remote and inaccessible nature for the community, making it challenging for the study team to reach and include patients from that area.

## 5. Conclusions

This study revealed suboptimal adherence to *P. falciparum* treatment in high malaria endemic areas of Bangladesh. However, studies should be conducted in the future where treatment adherence for *P. vivax* mono-infection would be extensively focused by strengthening a culturally suitable behaviour change communication strategy. Moreover, given issues with *P. vivax* treatment adherence, efforts on drug discovery with shorter treatment regimens are necessary. In order to explore community perceptions, attitudes, and practices regarding antimalarial treatment, qualitative research is recommended as well so that we can understand the corresponding socio-cultural factors that may impact adherence behaviours. So, it is crucial to integrate a robust health education system within the National Malaria Elimination Program (NMEP), ensuring that individuals of all age groups receive essential information. Regular monitoring and supportive supervision in hard-to-reach areas are also necessary to promote and maintain good treatment adherence, which, in turn, validates the cost-effective investment required to eliminate malaria from Bangladesh before 2030.

## Figures and Tables

**Table 1:** Baseline demographic characteristics of study participants (*n* = 110).

Characteristics	*Pf* Patient *n* (%)	*Pv* Patient *n* (%)	Total *n* (%)
**Upazila**			
Alikadam	36 (46.7)	15 (45.5)	51 (46.3)
Lama	30 (39.0)	10 (30.3)	40 (36.4)
Thanchi	5 (6.5)	3 (9.1)	8 (7.3)
Chakaria	6 (7.8)	5 (15.1)	11 (10.0)
**Sex**			
Male	56 (72.7)	28 (84.9)	84 (76.4)
Female	21 (27.3)	5 (15.1)	26 (23.6)
**Age, year**			
11–17 Years	30 (39.0)	7 (21.2)	37 (33.6)
18 Years and above	47 (61.0)	26 (78.8)	73 (66.4)
Median (IQR)	24 (14–38)	22 (18–28)	22 (15–36)
**Ethnicity**			
Mro	37 (48.0)	5 (15.1)	42 (38.2)
Tripura	15 (19.5)	5 (15.1)	20 (18.2)
Chakma	0 (0.0)	2 (6.1)	2 (1.8)
Marma	6 (7.8)	2 (6.1)	8 (7.3)
Tangchangya	3 (3.9)	2 (6.1)	5 (4.6)
Bengali	16 (20.8)	17 (51.5)	33 (30.0)
**Education**			
No formal education	34 (44.2)	8 (24.2)	42 (38.2)
Primary	25 (32.5)	9 (27.3)	34 (30.9)
Secondary	15 (19.5)	11 (33.3)	26 (23.6)
College/Graduate	3 (3.9)	5 (15.2)	8 (7.3)
**Occupation**			
Farming/Jhum Cultivation	34 (44.2)	8 (24.2)	42 (38.2)
Own business	3 (3.9)	3 (9.1)	6 (5.4)
Student	22 (28.6)	11 (33.3)	33 (30.0)
Daily labour	7 (9.1)	6 (18.2)	13 (11.8)
Housewife	4 (5.2)	0 (0.0)	4 (3.6)
Other	7 (9.1)	5 (15.2)	12 (10.9)
**Family Size (members)**			
≤4	16 (20.8)	14 (42.4)	30 (27.3)
≥5	61 (79.2)	19 (57.6)	80 (72.7)
**Respondent**			
Patient Self	50 (64.9)	25 (75.8)	75 (68.2)
Family member/Responsible for the child	27 (35.1)	8 (24.2)	35 (31.8)

**Table 2:** Level of adherence among the participants (*n* = 110).

Adherence Level	Nonadherent (NA)	Probably Adherent (PA)	Total	*p*-Value
	*n* (%)	*n* (%)		
*Plasmodium falciparum*	3 (3.9)	74 (96.1)	77	0.05
*Plasmodium vivax*	5 (15.1)	28 (84.9)	33	
Total	8 (7.3)	102 (92.7)	110	

## Data Availability

The data presented in this study are available from the corresponding author (M.S.A.) if requested reasonably.

## Supplementary Materials

The following supporting information can be downloaded at: https://www.mdpi.com/article/10.3390/pathogens12121392/s1, Table S1: Level of adherence among the participants (*n* = 110) by age, gender, and demographic location.
