# Supplementary material for: Adherence to Anti-Malarial Treatment in Malaria Endemic Areas of Bangladesh
Source: Pathogens. 2023 Nov 27;12(12):1392. doi: 10.3390/pathogens12121392 (PMC10745796; doi:10.3390/pathogens12121392)
Supplement: Supplementary file 1 [file pathogens-12-01392-s001.zip › pathogens-2724992-supplementary.pdf]

Supplementary Table

Table S1. Level of adherence among the participants (N = 110) by age, gender and demographic location.

Treatment group	Adherence level		Total, n	p-value
	**Non-adherent (NA), n (%)**	**Probably Adherent (PA), n (%)**		
	Age 11-17 Years, n=37			
Plasmodium falciparum	1 (3.3)	29 (96.7)	30	0.02
Plasmodium vivax	3 (42.9)	4 (57.1)	7	
	Age 18 Years and more, n=73			
Plasmodium falciparum	2 (4.3)	45 (95.7)	47	0.61
Plasmodium vivax	2 (7.7)	24 (92.3)	26	
	Male, n=84			
Plasmodium falciparum	3 (5.4)	53 (94.6)	56	0.40
Plasmodium vivax	3 (10.7)	25 (89.3)	28	
	Female, n=26			
Plasmodium falciparum	0 (0.0)	21 (100.0)	21	0.03
Plasmodium vivax	2 (40.0)	3 (60.0)	5	
	CHT area, n=99			
Plasmodium falciparum	3 (4.2)	68 (95.8)	71	0.04
Plasmodium vivax	5 (17.9)	23 (82.1)	28	
